# Establishment and validation of a nomogram based on coagulation parameters to predict the prognosis of pancreatic cancer

**DOI:** 10.1186/s12885-023-10908-0

**Published:** 2023-06-15

**Authors:** Peng Yunpeng, Yin Lingdi, Zhu Xiaole, Huang Dongya, Hu Le, Lu Zipeng, Zhang Kai, Hou Chaoqun, Miao Yi, Guo Feng, Li Qiang

**Affiliations:** 1 Pancreas Center, First Affiliated Hospital of Nanjing Medical University, 300 Guangzhou Road, Nanjing, 210029 Jiangsu Province People’s Republic of China; 2 Pancreas Institute, Nanjing Medical University, Nanjing, 210029 Jiangsu Province People’s Republic of China

**Keywords:** Pancreatic cancer, Coagulation and fibrinolysis parameters, Prognosis, Predictive nomogram

## Abstract

**Background:**

In recent years, multiple coagulation and fibrinolysis (CF) indexes have been reported to be significantly related to the progression and prognosis of some cancers.

**Objective:**

The purpose of this study was to comprehensively analyze the value of CF parameters in prognosis prediction of pancreatic cancer (PC).

**Methods:**

The preoperative coagulation related data, clinicopathological information, and survival data of patients with pancreatic tumor were collected retrospectively. Mann Whitney *U* test, Kaplan-Meier analysis, and Cox proportional hazards regression model were applied to analyze the differences of coagulation indexes between benign and malignant tumors, as well as the roles of these indexes in PC prognosis prediction.

**Results:**

Compared with benign tumors, the preoperative levels of some traditional coagulation and fibrinolysis (TCF) indexes (such as TT, Fibrinogen, APTT, and D-dimer) were abnormally increased or decreased in patients with pancreatic cancer, as well as Thromboelastography (TEG) parameters (such as R, K, α Angle, MA, and CI). Kaplan Meier survival analysis based on resectable PC patients showed that the overall survival (OS) of patients with elevated α angle, MA, CI, PT, D-dimer, or decreased PDW was markedly shorter than other patients; moreover, patients with lower CI or PT have longer disease-free survival. Further univariate and multivariate analysis revealed that PT, D-dimer, PDW, vascular invasion (VI), and tumor size (TS) were independent risk factors for poor prognosis of PC. According to the results of modeling group and validation group, the nomogram model based on independent risk factors could effectively predict the postoperative survival of PC patients.

**Conclusion:**

Many abnormal CF parameters were remarkably correlated with PC prognosis, including α Angle, MA, CI, PT, D-dimer, and PDW. Furthermore, only PT, D-dimer, and PDW were independent prognostic indicators for poor prognosis of PC, and the prognosis prediction model based on these indicators was an effective tool to predict the postoperative survival of PC.

**Supplementary Information:**

The online version contains supplementary material available at 10.1186/s12885-023-10908-0.

## Introduction

According to the latest studies, PC is still one of the most lethal malignancies originated in digestive system, and its 5-year survival rate is less than 10% [1]. Although researchers specialized in PC field have made considerable efforts in basic research, epidemiological analysis, diagnostic method, and treatment strategy in recent years, the current status of PC diagnosis and treatment is extremely frustrating [2]. Previous studies have shown that nearly 80% of PC patients had local progression or distant metastasis when they diagnosed. They had no opportunity to receive radical resection, and the survival of these patients was significantly shorter than patients receiving radical resection [3, 4]. Furthermore, even undergoing radical resection, the prognosis of PC patients also has significant differences. Therefore, it is of great significance to explore the factors which affecting the prognosis of PC patients with radical resection.

So far, an increasing number of prognostic factors of PC have been reported. For example, systemic immune inflammation index was defined as an independent risk factor for cancer specific survival and recurrence in resectable PC patients [5]; the infiltration levels of several lymphocyte phenotypes in microenvironment were closely related to the long-term oncological prognosis of patients with PC [6]; the abnormal expression of various tumor genes could predict the postoperative survival of PC (such as TRIM2, ALKBH5, and HHLA2) [7–9]. In addition, the roles of traditional coagulation and fibrinolysis (TCF) indexes in PC was gradually revealed. TT, APTT, PT, fibrinogen, and platelet related parameters were usually used as coagulation indicators in clinical practice, some of them were considered to be significantly correlated with PC prognosis. For example, patients with elevated platelet count had poor prognosis and high risk of distant metastasis [10, 11]; increased fibrinogen was an independent risk factor for shorter disease-free and overall survival in locally advanced PC [12]; serum fibrinogen was also an effective biomarker for PC diagnosis[13, 14]. D-dimer has also been reported to be markedly related to the PC progression and prognosis. For example, it has been reported that D-dimer could predict the resectability and 3-year survival of PC [15]; preoperative fibrinogen combined with D-dimer could be regarded as a predictor of overall survival in PC patients with R0 resection [16]. Compared with the TCF function test, TEG can monitor the coagulation process from the whole dynamic process of platelet aggregation, coagulation and fibrinolysis. After reviewing literature, there were relatively few studies focused on the correlation between TEG parameters and PC progression and prognosis. A clinical study found that preoperative TEG angle might be considered as a new biomarker for predicting early recurrence, disease-free survival and overall survival of pancreatic cancer [17]; another study suggested that the some indicators of TEG were significantly associated with pancreatic tumor malignancy evaluation, pancreatic cancer resectability, and nodal disease [18].

In view of the important roles of coagulation and fibrinolysis (CF) abnormalities in the prediction of PC progression and prognosis, this study intends to further verify whether the CF indicators could predict pancreatic cancer prognosis via a retrospective study; and establish an effective prediction model based on potential CF parameters.

## Materials and methods

### Patients

We retrospectively screened pancreatic tumors patients who received treatment in the pancreatic center of Jiangsu Province Hospital from June 2016 to June 2019. Inclusion criteria: patients with postoperative pathological diagnosis of pancreatic cancer (including ductal adenocarcinoma, adenocarcinoma, mucinous adenocarcinoma) and benign tumor (including serous cystadenoma, mucinous cystadenoma, and intraductal papillary mucinous tumor); simultaneously detected traditional CF and TEG indicators at admission; more than 18 years old. Exclusion criteria: patients with perioperative death (death within 30 days after surgery); received chemotherapy, radiotherapy, and immunotherapy before surgery; lack of necessary data (detailed clinicopathological data and/or follow-up data); long-term use of anticoagulants; combined with other diseases (malignant tumors, inflammatory disease, hematological diseases, cardiovascular disease, and cerebrovascular disease). The selection criteria of patients in validation group was similar to modeling group; however, the patients in validation group only need traditional CF data at admission, and overall survival data. This study has been approved by the ethics committee of Jiangsu Province Hospital, and informed consent has been signed with all patients.

### Data Collection

All clinicopathological and follow-up data were prospectively collected by the clinical database of our center. The clinicopathological data included age, gender, preoperative traditional CF indicators (including TT, APTT, PT, FIB, D-dimer, PLT, PCT, PDW, and MPV). TT, APTT, PT, D-dimer, and FIB were obtained from coagulation test; PLT, PCT, PDW, and MPV were obtained from complete blood count. Preoperative TEG parameters (including R, K, α angle, MA, CI, and Ly30) were obtained from Thrombelastograph Hemostasis System. Postoperative pathological data (including pathological diagnosis, tumor location, tumor size, histological grade, T stage, N stage, M stage, TNM stage, vascular invasion) were also collected. The follow-up data included the disease-free survival (DFS) and overall survival, and the last follow-up date was April 15, 2021. Disease-free survival (DFS) was defined as the time interval between the date of surgery and either date of recurrence or death, which came first or censored at last follow-up, and recurrence was evaluated with CT or MR. According to the follow-up data, 40 (40/101) patients were alive and 37 (37/88) patients were disease-free in modeling group; 31 (31/101) patients were alive in validation group. All the data applied in this study were further reviewed by two independent researchers.

### Statistical analysis

Continuous variables were expressed as means ± SD. Mann Whitney U test was used to compare the differences between two groups, while Chi-square test was applied to compare the differences of categorical variables between two groups. Survminer and X-tile were used to calculate the cut-off value of the continuous variables. Kaplan-Meier method and log rank t test were used to compare survival differences between groups and draw survival curves. Univariate and multivariate analysis based on Cox proportional hazards region model were applied to identify independent risk factors, and variables with p values less than 0.1 were included for multivariate analysis. The method of predictive nomogram construction and validation were provided in previous study published by Mengwei Wu et al. [19]. p value less than 0.05 was defined as statistically significant.

## Results

### Patient characteristics

A total of 164 patients were finally included in modeling group. Among them, 130 patients were pathologically diagnosed as pancreatic cancer (including 101 patients received radical resection; 29 patients with distant metastasis and/or local progression underwent tumor biopsy and/or palliative internal drainage). 34 patients were pathologically diagnosed as benign pancreatic tumors (including 12 cases of intraductal papillary mucinous tumors, 8 cases of pancreatic mucinous cystadenoma and 14 cases of pancreatic mucinous cystadenoma), and all of them received resection. According to the pathological diagnosis and operation procedure, all patients were divided into three groups, including benign tumor group, resectable PC group, and unresectable PC group. The clinical information of patients in different groups was shown in Table 1.

**Table 1:** The clinical information of enrolled patients with pancreatic tumor

	BPT	RPC	URPC
Number	34	101	29
Age	61(28–81)	65(33–81)	62(48–80)
Gender			
Male	16	63	18
Female	18	38	11
Location			
Head/ Head & Neck	12	65	12
Neck/Neck& Body /Body/Tail	22	36	17
TNM stage			
IA		6	
IB		25	
IIA		9	
IIB		42	
III		18	7
IV		1	22

BPT represent as benign pancreatic tumor, RPC represent as resectable pancreatic cancer, URPC represent as unresectable pancreatic cancer

### The association between CF indicators and pancreatic tumor types

We firstly analyzed the differences of CF parameters between benign and malignant tumors. The results revealed that malignant pancreatic tumor patients had obvious abnormalities of CF system, which represent as some CF parameters abnormally increased or decreased (including R, K,α Angle, MA, CI, TT, fibrinogen, APTT, and D-dimer) (Table 2). PC Patients were divided into resectable and unresectable groups, and subgroup analysis showed that not only resectable but also unresectable PC patients had obvious hypercoagulation state and secondary hyperfibrinolysis compared to benign tumor patients; however, only PT and D-dimer were further increased in unresectable patients (Table 3). These results suggested that many CF parameters might be valuable in differentiating benign and malignant pancreatic tumors; however, the diagnostic value of these parameters need to be further evaluated and verified.

**Table 2:** The difference of CF parameters between benign and malignant pancreatic tumors

	BPT(N = 34)		MPT(N = 130)		*P* value
	Mean	SD	Mean	SD	
R (min)	5.69	1.04	5.24	1.14	0.013
K (min)	2.06	0.75	1.80	0.74	0.015
αAngle (deg)	63.33	6.49	66.53	7.08	0.007
MA (mm)	60.84	5.08	62.91	6.88	0.047
CI	-0.03	1.65	0.86	1.85	0.005
Ly30 (%)	1.17	3.20	0.88	2.59	0.598
TT (s)	18.92	3.86	17.80	1.11	0.017
FIB (g/L)	2.86	0.99	3.81	1.18	0.000
APTT (s)	28.01	3.16	26.78	2.76	0.035
PT (s)	11.88	0.75	11.94	0.77	0.317
D-dimer (mg/L)	0.62	1.19	0.94	1.62	0.001
PLT (10^9/L)	199.00	63.42	200.62	78.56	0.995
PCT (%)	0.21	0.06	0.22	0.09	0.629
PDW (%)	15.63	2.84	16.06	2.27	0.235
MPV (fL)	11.09	1.62	10.83	1.76	0.303

**Table 3:** Subgroup analysis of CF parameter difference between different pancreatic tumor types

	BPT(N = 34)		RPC(N = 101)		URPC(N = 29)		P^*^ value	*P*^*#*^ value	*P*^&^ value
	Mean	SD	Mean	SD	Mean	SD			
R (min)	5.69	1.04	5.25	1.20	5.23	0.91	0.017	0.046	0.724
K (min)	2.06	0.75	1.83	0.79	1.71	0.52	0.019	0.060	0.980
αAngle (deg)	63.33	6.49	66.39	7.44	67.00	5.78	0.009	0.044	0.887
MA (mm)	60.84	5.08	62.77	7.14	63.37	5.99	0.058	0.111	0.973
CI	-0.03	1.65	0.82	1.94	0.99	1.52	0.009	0.020	0.958
Ly30 (%)	1.17	3.20	1.00	2.89	0.49	0.90	0.382	0.500	0.187
TT (s)	18.92	3.86	17.86	1.07	17.57	1.23	0.033	0.020	0.230
FIB (g/L)	2.86	0.99	3.80	1.21	3.83	1.09	0.000	0.000	0.806
APTT (s)	28.01	3.16	26.76	2.87	26.84	2.41	0.031	0.195	0.450
PT (s)	11.88	0.75	11.84	0.78	12.31	0.57	0.845	0.004	0.000
D-dimer (mg/L)	0.62	1.19	0.88	1.74	1.12	1.12	0.003	0.000	0.037
PLT (10^9/L)	199.00	63.42	204.64	79.90	186.59	73.32	0.755	0.385	0.213
PCT (%)	0.21	0.06	0.22	0.09	0.20	0.09	0.955	0.136	0.159
PDW (%)	15.63	2.84	16.14	2.18	15.75	2.57	0.211	0.530	0.920
MPV (fL)	11.09	1.62	10.86	1.83	10.71	1.55	0.399	0.227	0.497

P^*^ represent as BPT *VS* RPC, *P*^*#*^ represent as BPT *VS* URPC, *P*^&^ represent as RPC *VS* URPC.

### The correlation between CF parameters and pancreatic cancer prognosis and progression

**Fig. 1:**
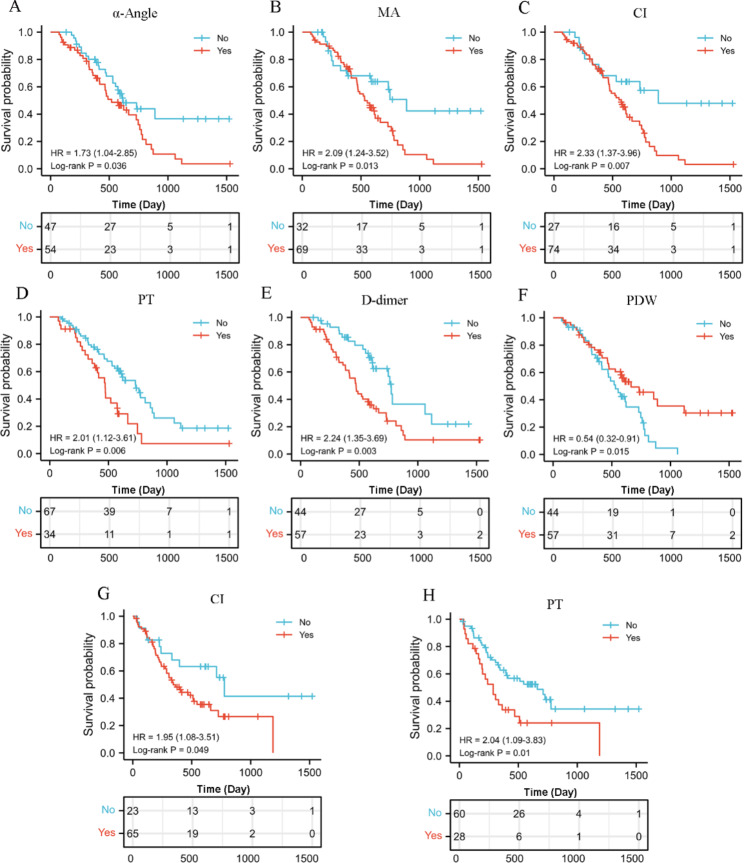
The association between CF parameters and pancreatic cancer prognosis. (**A**-**F**) The association between CF parameters (including α angle, Ma, CI, PT, D-dimer, and PDW) and overall survival of PC. (**G**, **H**) The association between CF parameters (including CI, and PT) and disease-free survival of PC.

The optimal cut-off values of each CF index was shown in the Supplementary Table 1. According to the optimal cut-off value, PC patients were divided into two groups, and the correlation between the indicators and patient survival was further analyzed. As shown in the Fig. 1A-F, and Supplementary Tables 2, some TEG parameters, such as α angle, MA, and CI, were negatively associated with the overall survival of PC patients, which presented as the overall survival time of patients with higher α angle, MA, or CI levels was significantly longer than that of patients with lower levels; several TCF indicators also had definite relationships with the overall survival. The trend of PT and D-dimer was similar to α angle, MA, and CI, while the trend of PDW was opposite to them. In addition, explorations focused on DFS showed that CI and PT were negatively correlated with DFS (Fig. 1G-H, and Supplementary Table 3). Compared to patients with lower level of CI or D-dimer, patients with higher level of CI or D-dimer had shorter DFS. These results indicate that both TEG parameters and TCF parameters could potentially predict the prognosis of patients with PC.

Furthermore, the analytic data revealed that MA and D-dimer were markedly associated with certain progression indicators, such as the MA or D-dimer level of poorly differentiated PC patients was significantly higher than Well differentiated patients; and the bigger tumor size was respectively with lower D-dimer content. These results suggested the abnormity of partial CF parameters could indicate the PC progression (Supplementary Table 4).

### The construction and validation of prognostic nomogram for PC

**Fig. 2:**
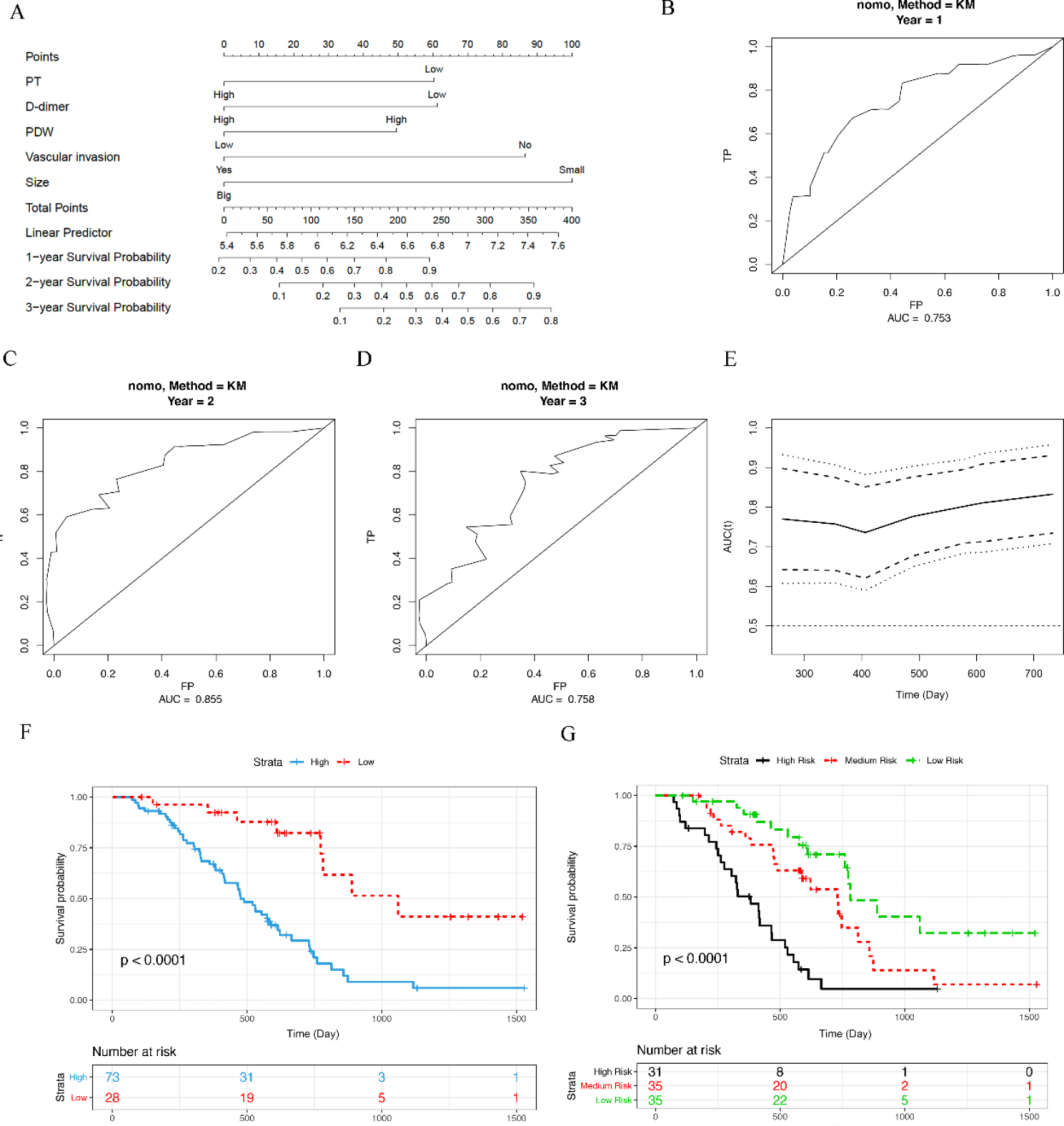
The Construction of prognostic nomogram for PC. (**A**) The prognostic nomogram for PC based on PT, D-dimer, PDW, VI, and TS. (**B**-**D**) The time-dependent ROC for 1-, 2-, and 3-year overall survival predictions. (**E**) The time dependent AUC of the nomogram in predicting PC overall survival. (**F**, **G**) The survival analysis of the nomogram. All patients were divided into two or three group according to optimal cutoffs provided by Survminer

**Fig. 3:**
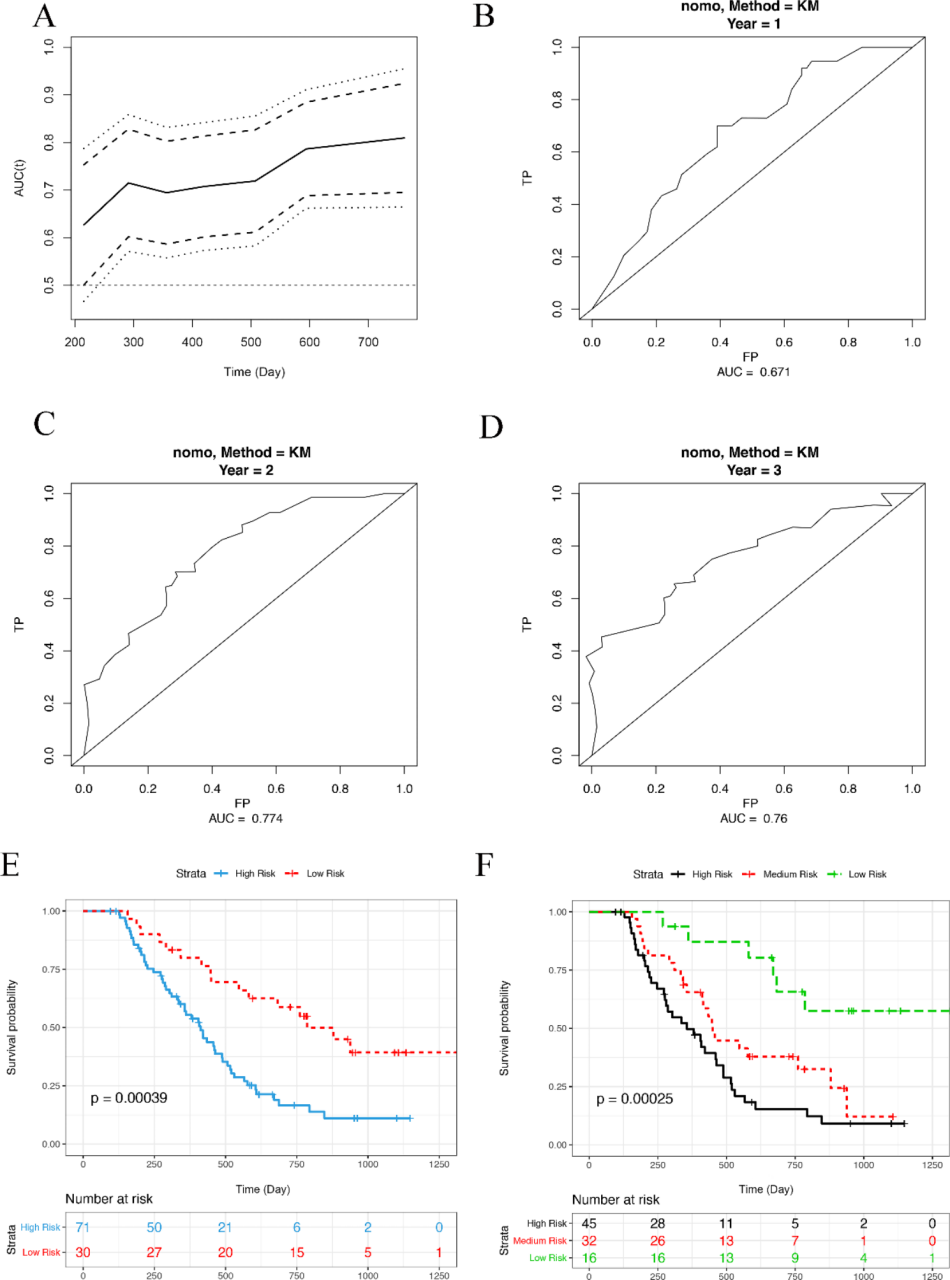
The Validation of prognostic nomogram for PC. (**A**) The time dependent AUC of validation cohort in predicting PC overall survival. (**B**-**D**) The time-dependent ROC for 1-, 2-, and 3-year overall survival predictions of validation cohort. (**E**, **F**) The survival analysis based on data from validation cohort. All patients were divided into two or three group according to optimal cutoffs provided by Survminer

We further analyzed independent risk factors by using Cox proportional hazards regression model. The analytic results showed that PT, D-dimer, and PDW were independent risk factors for poor prognosis of PC (Table 4). In addition, PT, D-dimer, PDW, VI and TS were used to establish a prognostic nomogram for PC. The results showed that the nomogram could effectively predict the PC prognosis, of which the the AUCs of the 1-, 2-, and 3-year OS predictions were 0.735, 0.855, and 0.758, respectively, and the C-index of the risk score was 0.735 (Fig. 2A-E). According to the nomogram score, PC patients were divided into two or three group, and the OS of patients with lower risk was significantly better than patients with higher risk (Fig. 2F, G). In order to further verify the efficiency of this nomogram, another 101 PC patients (Supplementary Table 5) were enrolled as the validation cohort. The trend of following results was consistent with that in the modeling cohort. The AUCs of the 1-, 2-, and 3-year OS predictions in validation group were 0.671, 0.774, and 0.760. And the C-index of the risk score was 0.656 (Fig. 3A-D). The OS of patients with lower scores in the validation group was longer than patients with higher scores (Fig. 3E, F). All these data revealed that the prognosis model based on above-mentioned parameters was an effective tool to predict the postoperative survival of PC.

**Table 4:** Univariate and multivariate analysis by using Cox proportional hazards regression model

	Univariate Analysis			Multivariate Analysis		
	HR	95% CI	P	HR	95% CI	P
R (≤ 4.6 VS >4.6)	0.74	0.44–1.23	0.239			
K (≤ 1.6 VS >1.6)	0.61	0.36–1.01	0.054	1.23	0.38–3.98	0.731
αAngle (≤ 67 VS >67)	1.74	1.03–2.92	0.038	1.26	0.37–4.26	0.709
MA (≤ 60.4 VS >60.4)	2.17	1.16–4.06	0.016	1.17	0.44–3.09	0.758
CI (≤-0.3 VS >-0.3)	2.44	1.25–4.77	0.009	1.06	0.33–3.36	0.921
Ly30 (0 VS >0)	1.41	0.84–2.34	0.191			
TT (≤ 18.1 VS >18.1)	0.70	0.40–1.21	0.200			
FIB (≤ 3.03 VS >3.03)	1.38	0.76–2.51	0.294			
APTT (≤ 26.3 VS >26.3)	0.71	0.43–1.18	0.186			
PT (≤ 12 VS >12)	2.08	1.23–3.52	0.007	2.00	1.12–3.57	0.019
D-dimer (≤ 0.4 VS >0.4)	2.251	1.31–3.88	0.003	1.84	1.03–3.29	0.041
PLT (≤ 194 VS >194)	0.722	0.44–1.20	0.208			
PCT (≤ 0.19 VS >0.19)	0.84	0.50–1.40	0.493			
PDW (≤ 16.3 VS >16.3)	0.53	0.32–0.89	0.016	0.51	0.27–0.96	0.038
MPV (≤ 10.11 VS >10.11)	1.73	0.93–3.20	0.082	1.20	0.59–2.44	0.623
TD (1/1–2/2 VS 2–3 VS 3/4 )	1.27	0.86–1.87	0.225			
TS (≤ 2.5 VS >2.5)	2.66	1.37–5.19	0.004	2.41	1.15–5.06	0.020
T stage (T1/T2 VS T3/T4)	1.41	0.83–2.37	0.204			
N stage (N0 VS N1 VS N2)	1.27	0.88–1.83	0.195			
TNM (I VS II VS III/IV)	1.31	0.92–1.88	0.139			
VI (No VS Yes)	1.90	1.15–3.16	0.013	2.34	1.34–4.07	0.003

## Discussion

In this study, we found that the levels of many CF indexes were different between benign and malignant pancreatic tumors. Although multiple TEG or TCF parameters were closely associated with the PC prognosis, only PT, D-dimer, and PDW were independent risk factors for PC overall survival prediction. The data of modeling group and validation group showed that predictive nomogram based on PT, D-dimer, and PDW could effectively predict the overall survival of PC.

In recent years, it has been reported that conventional coagulation components played important roles in many malignant biological behaviors. For example, GP Ib-IX-V complex on platelet surface could bind to integrin on circulating blood tumor cells through fibrinogen αvβ3 to further promote the distant metastasis of tumor [20, 21]; activation of platelets by TGF-β could inhibit the killing activity of NK cells [22]; platelets could also significantly repress T cell proliferation via GARP/TGF-β pathway, as well as blastogenesis and IFN-γ expression [23]. In view of the highly participatory of coagulation components in tumor progression, some researchers evaluated the possibility of coagulation indicators as prognostic indicators for cancer patients. Related studies have shown that some coagulation indicators could acted as prognostic predictive biomarkers. For example, PT could be used as a prognostic predictive biomarker for postoperative recurrence in stage I-III colorectal cancer patients[24]; abnormally increased PT could serve as a simple but effective prognostic predictor for cholangiocarcinoma patients with curative resection[25]; lower preoperative PDW levels were observed in hepatocellular carcinoma patients with pulmonary metastasis, and PDW could work as an independent predictor for pulmonary metastasis[26]; disease-free survival and overall survival of non-small cell lung cancer patients with PDW ≤ 12.65 were both significantly shorter than patients with PDW > 12.65 [27]; decreased PDW was a poor prognostic factor for patients with early colon cancer, especially stage III patients[28]. The results of our study were similar to those of previous studies, which also verify that many traditional coagulation indexes were abnormal in PC; among them, PT and PDW had independent predictive value for PC prognosis.

Additionally, conventional fibrinolytic indexes also plays a role in tumor progression. Previous studies have shown that tumor cell-derived t-PA and u-PA could activate plasminogen to form plasmin, and then accelerate local invasion and distant metastasis of tumor cells [29–31]; T-PA and u-PA were also proved to be abnormally elevated in the plasma of patients with malignant tumor, and they were closely related to the resectability and shortened survival time [32, 33]. Similar to T-PA and u-PA, D-dimer could also act as an prognostic predictor for certain cancer patients. For example, pretreatment elevated D-dimer could deserve as a reliable biomarker to predict prognosis of patients with small cell lung cancer[34]; increased preoperative plasma levels of D-dimer were significantlly associated with chemoresistance and poor prognosis in patients with serous ovarian cancer[35]; D-dimer was also abnormally increased in pancreatic cancer patients, and the concentration of this index was remarkablely correlated with the poor outcome of pancreatic [15, 36]. Our results also showed that D-dimer had significant predictive value for overall survival and disease-free survival of pancreatic cancer, and D-dimer was also an independent risk factor for poor prognosis of PC.

Besides TCF indexes, TEG also has the value of dynamic evaluation of coagulation function, related indicators contain R, K, α angle, MA, and CI [37]. In previous cancer-related studies, TEG was often used as a tool for dynamic detection of perioperative coagulation function [38, 39], as a predictor of venous thrombosis and postoperative bleeding [40, 41], or a predictive biomarker for cancer progression [42, 43]. However, there were few studies focused on the correlation between these indicators and PC development [17, 18]. In this study, we innovatively found that abnormally altered α angle, MA, and CI were identified as prognostic factors for PC; however, all these parameters were not independent risk factors according to the multivariate analysis results.

With the innovation of statistical methods, the method of using single factor to predict tumor prognosis has gradually been replaced by nomogram which integrating multiple factors. According to the results of previous studies, the prediction efficiency of nomogram for cancer diagnosis was significantly higher than that of single factor integrated into the model [44]. Wang et al. combined three lncRNAs, TNM stage, and age to construct a nomogram for predicting prognosis of bladder cancer, and the model worked better than the lncRNAs signature or clinical factors alone for survival prediction [45]; Wu et al. proved that nomogram constructed by nine gene signature and clinical factors could reliably predict the prognosis of PC [19]. Refering to methods mentioned above, we innovatively constructed an overall survival related nomogram for PC patients based on the data of PT, D-dimer, and PDW, and the subsequent results suggested that this model had favourable predictive power for PC prognosis. And then, the verification result based on validation cohort further confirmed the predictive efficiency of this nomogram for the outcome of PC. The selected factors for this prognostic nomogram was conventionally detected in clinical practice without additional cost, the acquisition of related data was extremely convenient. Considering the accessibility, economy, and efficiency, our nomogram model would have a broad prospect for clinical application.

Moreover, the study also had some limitations. Firstly, all factors and prognosis data applied in this study were obtained from 101 patients with PC, while the relatively small sample size might lead to the omission of some potential prognostic factors. Secondly, the study did not include some other factors influencing PC prognosis, such as postoperative radiotherapy and chemotherapy, postoperative Chinese medicine treatment, and postoperative nutritional status. In addition, the lack of external data verification in this study affects the level of evidence of the results to some extent. In the future, we will carry out a prospective large sample study with larger sample size to verify the prognostic roles of CF parameters in PC, and further modify the related nomogram.

## Conclusion

This study comprehensively analyzed the correlation between various coagulation parameters and PC prognosis, and further constructed a prognosis related nomogram based on PT, D-dimer, PDW, and clinicopathological factors. The prognostic nomogram effectively predicted PC overall survival, and provides a theoretical basis for individualized treatment of PC.

## Electronic supplementary material

Below is the link to the electronic supplementary material.

Supplementary Material 1

## Data Availability

All data applied in this study were provided in Related files.

## Abbreviations

CF: Coagulation and fibrinolysis
PC: Pancreatic cancer
TCF: Traditional coagulation and fibrinolysis
FIB: Fibrinogen
TT: Thrombin time
APTT: Activated partial thromboplastin time
PT: Prothrombin time
PDW: Platelet distribution width
PLT: Platelet count
PCT: Platelet hematocrit
MPV: Mean platelet volume
TEG: Thromboelastography
R: Reaction time
K: Clot formation time
MA: Maximum amplitude
CI: Coagulation index
BPT: Benign pancreatic tumor
MPT: Malignant pancreatic tumor
RPC: Resectable pancreatic cancer
URPC: Unresectable pancreatic cancer
VI: Vascular invasion
TS: Tumor size
TD: Tumor differentiation
