# Supplementary material for: Establishment and validation of a nomogram based on coagulation parameters to predict the prognosis of pancreatic cancer
Source: BMC Cancer. 2023 Jun 15;23:548. doi: 10.1186/s12885-023-10908-0 (PMC10273637; doi:10.1186/s12885-023-10908-0)
Supplement: Supplementary file 1 — Supplementary Material 1 [file 12885_2023_10908_MOESM1_ESM.doc]

**Supplementary Table 1. Cut-off value for CF parameters**

| CF parameters | Cut-off value |
| --- | --- |
| R | 4.6 |
| K | 1.6 |
| αAngle | 67 |
| MA | 60.4 |
| CI | -0.3 |
| Ly30 | 0 |
| TT | 18.1 |
| FIB | 3.03 |
| APTT | 26.3 |
| PT | 12 |
| D-dimer | 0.4 |
| PLT | 194 |
| PCT | 0.19 |
| PDW | 16.3 |
| MPV | 10.11 |

**Supplementary Table 2. The correlation between CF parameters and pancreatic cancer overall survival**

| CF parameters | Chi-Square | P value |
| --- | --- | --- |
| R | 1.4 | 0.237 |
| K | 3.798 | 0.051 |
| αAngle | 4.416 | 0.036 |
| MA | 6.115 | 0.013 |
| CI | 7.249 | 0.007 |
| Ly30 | 1.725 | 0.189 |
| TT | 1.662 | 0.197 |
| FIB | 1.109 | 0.292 |
| APTT | 1.768 | 0.184 |
| PT | 7.684 | 0.006 |
| D-dimer | 9.012 | 0.003 |
| PLT | 1.599 | 0.206 |
| PCT | 0.47 | 0.493 |
| PDW | 5.964 | 0.015 |
| MPV | 3.09 | 0.079 |

**Supplementary Table 3. The correlation between CF parameters and pancreatic cancer disease free survival**

| CF parameters | Chi-Square | P value |
| --- | --- | --- |
| R | 0.195 | 0.659 |
| K | 0.529 | 0.467 |
| αAngle | 2.326 | 0.127 |
| MA | 0.541 | 0.462 |
| CI | 3.886 | 0.049 |
| Ly30 | 0.573 | 0.449 |
| TT | 2.512 | 0.113 |
| FIB | 0.405 | 0.525 |
| APTT | 0.043 | 0.836 |
| PT | 6.648 | 0.01 |
| D-dimer | 2.341 | 0.126 |
| PLT | 3.038 | 0.081 |
| PCT | 2.248 | 0.134 |
| PDW | 0.732 | 0.392 |
| MPV | 0.559 | 0.455 |

**Supplementary Table 4. The correlation between CF parameters and clinicopathologic characteristics of pancreatic cancer**

|  | | αAngle | | MA | | CI | | PT | | D-dimer | | PDW | |
| --- | --- | --- | --- | --- | --- | --- | --- | --- | --- | --- | --- | --- | --- |
|  |  | ≤67 | ＞67 | ≤60.4 | ＞60.4 | ≤-0.3 | ＞-0.3 | ≤12 | ＞12 | ≤0.4 | ＞0.4 | ≤16.3 | ＞16.3 |
| T stage | T1/T2 | 35 | 34 | 22 | 47 | 21 | 48 | 47 | 22 | 33 | 36 | 28 | 41 |
|  | T3/T4 | 12 | 20 | 10 | 22 | 6 | 26 | 20 | 12 | 11 | 21 | 16 | 16 |
|  | *P* |  | 0.215 |  | 0.949 |  | 0.217 |  | 0.578 |  | 0.205 |  | 0.374 |
| N stage | N0 | 17 | 23 | 12 | 28 | 9 | 31 | 25 | 15 | 17 | 23 | 17 | 23 |
|  | N1 | 21 | 24 | 15 | 30 | 13 | 32 | 32 | 13 | 21 | 24 | 21 | 24 |
|  | N2 | 9 | 7 | 5 | 11 | 5 | 11 | 10 | 6 | 6 | 10 | 6 | 10 |
|  | *P* |  | 0.648 |  | 0.946 |  | 0.726 |  | 0.661 |  | 0.805 |  | 0.805 |
| TNM | IA/IB | 12 | 19 | 8 | 23 | 8 | 23 | 20 | 11 | 13 | 18 | 12 | 19 |
|  | IIA/IIB | 26 | 25 | 19 | 32 | 14 | 37 | 34 | 17 | 24 | 27 | 24 | 27 |
|  | III/IV | 9 | 10 | 5 | 14 | 5 | 14 | 13 | 6 | 7 | 12 | 8 | 11 |
|  | *P* |  | 0.556 |  | 0.477 |  | 0.986 |  | 0.958 |  | 0.728 |  | 0.753 |
| Differentiation | 1/1-2/2 | 19 | 22 | 19 | 22 | 12 | 29 | 27 | 14 | 23 | 18 | 15 | 26 |
|  | 2-3 | 23 | 24 | 12 | 35 | 13 | 34 | 33 | 14 | 19 | 28 | 24 | 23 |
|  | 3/4 | 5 | 8 | 1 | 12 | 2 | 11 | 7 | 6 | 2 | 11 | 5 | 8 |
|  | *P* |  | 0.798 |  | 0.015 |  | 0.604 |  | 0.541 |  | 0.03 |  | 0.363 |
| Vascular Invasion | No | 31 | 32 | 23 | 40 | 19 | 44 | 40 | 23 | 29 | 34 | 27 | 36 |
|  | Yes | 16 | 22 | 9 | 29 | 8 | 30 | 27 | 11 | 15 | 23 | 17 | 21 |
|  | P |  | 0.488 |  | 0.18 |  | 0.316 |  | 0.436 |  | 0.52 |  | 0.854 |
| Tumor Size (cm) | ≤2.5 | 11 | 15 | 10 | 16 | 10 | 16 | 18 | 8 | 16 | 10 | 14 | 12 |
|  | ＞2.5 | 36 | 39 | 22 | 53 | 17 | 58 | 49 | 26 | 28 | 47 | 30 | 45 |
|  | *P* |  | 0.616 |  | 0.389 |  | 0.117 |  | 0.717 |  | 0.032 |  | 0.22 |

**Supplementary Table 5. The clinicopathologic information of pancreatic cancer patients enrolled in validation cohort**

|  | RPC |
| --- | --- |
| Number | 101 |
| Age | 64(43-84) |
| Gender |  |
| Male | 57 |
| Female | 44 |
| Location |  |
| Head/ Head & Neck | 61 |
| Neck/Neck& Body /Body/Tail | 38 |
| Head & Neck & Body | 2 |
| TNM stage |  |
| IA | 3 |
| IB | 20 |
| IIA | 4 |
| IIB | 44 |
| III | 26 |
| IV | 4 |

**Supplementary Table 6. The minimum, maximum, and median of variables that did not conform to a normal distribution.**

|  | Minimum | Maximum | Median |
| --- | --- | --- | --- |
| Ly30 in URPC | 0 | 3 | 0 |
| FIB in URPC | 2.25 | 6.3 | 3.39 |
| D-dimer in URPC | 0.17 | 4.31 | 0.67 |
| PCT in URPC | 0.09 | 0.39 | 0.17 |
| PDW in URPC | 10.9 | 20 | 16.5 |
| K in BPT | 1.2 | 4.6 | 1.8 |
| αAngle in BPT | 43.6 | 71.7 | 65.65 |
| Ly30 in BPT | 0 | 13.1 | 0 |
| TT in BPT | 15.7 | 39.8 | 18.25 |
| FIB in BPT | 1.86 | 6.37 | 2.425 |
| PT in BPT | 10.8 | 13.7 | 11.5 |
| D-dimer in BPT | 0.1 | 6.51 | 0.32 |
| PLT in BPT | 99 | 381 | 180 |
| PCT in BPT | 0.11 | 0.38 | 0.2 |
| R in RPC | 2.8 | 8.8 | 5 |
| K in RPC | 0.8 | 5 | 1.7 |
| αAngle in RPC | 41.6 | 80.1 | 67.5 |
| Ly30 in RPC | 0 | 21.2 | 0 |
| FIB in RPC | 1.66 | 6.92 | 3.51 |
| APTT in RPC | 19.6 | 35.6 | 26.7 |
| PT in RPC | 10.3 | 14.5 | 11.8 |
| D-dimer in RPC | 0.1 | 15.72 | 0.46 |
| PLT in RPC | 49 | 457 | 190 |
| PCT in RPC | 0.05 | 0.54 | 0.2 |
| PDW in RPC | 11.1 | 23.7 | 16.5 |
| R in MPT | 2.8 | 8.8 | 5.05 |
| K in MPT | 0.8 | 5 | 1.7 |
| Ly30 in MPT | 0 | 21.2 | 0 |
| TT in MPT | 15.6 | 22.6 | 17.8 |
| FIB in MPT | 1.66 | 6.92 | 3.49 |
| D-dimer in MPT | 0.1 | 15.72 | 0.505 |
| PLT in MPT | 49 | 457 | 186.5 |
| PCT in MPT | 0.05 | 0.54 | 0.195 |
| PDW in MPT | 10.9 | 23.7 | 16.525 |
| MPV in MPT | 7.4 | 17.3 | 10.835 |
